# Anthropometric indices of Gambian children after one or three annual rounds of mass drug administration with azithromycin for trachoma control

**DOI:** 10.1186/1471-2458-14-1176

**Published:** 2014-11-18

**Authors:** Sarah E Burr, John Hart, Tansy Edwards, Emma M Harding-Esch, Martin J Holland, David C W Mabey, Ansumana Sillah, Robin L Bailey

**Affiliations:** Department of Clinical Research, London School of Hygiene and Tropical Medicine, London, UK; Disease Control and Elimination Theme, Medical Research Council Unit, The Gambia, Fajara, Banjul, The Gambia; MRC Tropical Epidemiology Group, Department of Infectious Disease Epidemiology, London School of Hygiene and Tropical Medicine, London, UK; HIV/STI Department, Colindale, Public Health England, London, UK; National Eye Health Programme, Ministry of Health, Kanifing, The Gambia

**Keywords:** Mass drug administration, Azithromycin, Trachoma control, Anthropometry

## Abstract

**Background:**

Mass drug administration (MDA) with azithromycin, carried out for the control of blinding trachoma, has been linked to reduced mortality in children. While the mechanism behind this reduction is unclear, it may be due, in part, to improved nutritional status via a potential reduction in the community burden of infectious disease. To determine whether MDA with azithromycin improves anthropometric indices at the community level, we measured the heights and weights of children aged 1 to 4 years in communities where one (single MDA arm) or three annual rounds (annual MDA arm) of azithromycin had been distributed.

**Methods:**

Data collection took place three years after treatment in the single MDA arm and one year after the final round of treatment in the annual MDA arm. Mean height-for-age, weight-for-age and weight-for-height *z* scores were compared between treatment arms.

**Results:**

No significant differences in mean height-for-age, weight-for-age or weight-for-height *z* scores were found between the annual MDA and single MDA arms, nor was there a significant reduction in prevalence of stunting, wasting or underweight between arms.

**Conclusions:**

Our data do not provide evidence that community MDA with azithromycin improved anthropometric outcomes of children in The Gambia. This may suggest reductions in mortality associated with azithromycin MDA are due to a mechanism other than improved nutritional status.

## Background

Targeted disease control interventions often have broader effects on child nutrition and mortality. For example, vaccination with measles and Bacille Calmette Guerin (BCG) vaccines are reported to be associated with reductions in mortality that cannot be explained solely by the prevention of measles and tuberculosis respectively [1, 2]. Reductions in child mortality have also been associated with mass drug administration (MDA) with vitamin A, given for the prevention of childhood blindness [3]. Other benefits of targeted interventions include improved growth and cognitive function and reductions in intestinal permeability associated with MDA with albendazole given for the treatment of helminth infection [4–6].

MDA with the broad-spectrum antibiotic azithromycin is a corner-stone of the World Health Organization-recommended strategy for control of ocular *Chlamydia trachomatis* infection, the etiological agent of blinding trachoma [7, 8]. Current WHO guidelines recommend three annual rounds of MDA with azithromycin in districts where the prevalence of follicular trachoma (TF) is ≥10% in children aged 1 to 9 years, with treatment coverage of at least 80% [9]. Recommended treatment at each round of MDA is a single oral dose of azithromycin given at 20 mg/kg to a maximum of 1 g. In Ethiopia, overall mortality among 1–9 year olds has reportedly been reduced by 49% in communities receiving intermittent MDA with azithromycin for trachoma control [10, 11]. The publication of these findings has since lead to a call to further investigate the impact of such MDA on broader child health measures including anthropometry [12].

While the mechanism underlying the remarkable reduction in mortality reported in Ethiopia is not yet clear, it may be due, in part, to improved nutritional status among children receiving azithromycin. A recent systematic review of trials looking at associations of antibiotic use and growth has suggested that antibiotic use promotes both linear growth and weight gain in children in low and middle-income countries [13]. Diarrhoeal disease and lower respiratory tract infection are associated with growth faltering in many low income countries [14–17] and evidence suggests that MDA with azithromycin reduces the incidence of both respiratory and gastrointestinal infections [18–20]. It is therefore plausible that a reduced burden of infectious disease following MDA leads to improved growth. Environmental enteropathy has also been shown to contribute to growth faltering in several African and Asian countries [21–24]. This condition, thought to be caused by chronic exposure to faecal bacteria, leads to microbial translocation and chronic immune activation [21, 25]. Modulation of the intestinal microbiota, as a result of azithromycin’s antibacterial action, or a direct reduction in immune activation via the immunomodulatory effects of azithromycin [26] may also have the potential to improve an individual’s growth.

The Partnership for the Rapid Elimination of Trachoma (PRET) trial was designed to measure the cost-effectiveness of approaches to mass treatment with azithromycin for the control of trachoma [27–30] and provided an opportunity to investigate improved nutritional status as a possible mechanism for the impact on mortality attributed to azithromycin. To this end, we carried out an ancillary study to collect height and weight measurements of children in the PRET trial communities in The Gambia, West Africa. The aims were to compare anthropometric indices of children, aged 1–4 years, in communities randomized to receive three annual rounds of mass azithromycin treatment (annual MDA arm) to those of children in communities where a single round of mass treatment was given at baseline (single MDA arm).

## Methods

### Ethics statement

This study was ancillary to the PRET trial and was approved by The Gambia Government/Medical Research Council Unit, The Gambia Joint Ethics Committee. Informed, written consent was obtained from each child’s guardian at the time of examination. The consent form was also signed by an independent witness.

### Study design

The PRET study (ClinicalTrials.gov NCT00792922) was a cluster randomized trial to evaluate coverage and frequency of mass treatment with azithromycin on the prevalence of active trachoma and ocular *C. trachomatis* infection. A detailed description of the trial design has previously been published [27]. Briefly, clusters, or enumerations areas (EAs, population 600–800 individuals), were randomly sampled from four districts in The Gambia, West Africa (12 EAs per district). Twenty-four EAs received three annual rounds of MDA with azithromycin as per current WHO recommendations (annual MDA arm: MDA at baseline, year one and year two) and 24 EAs received a single round of MDA at baseline (single MDA arm). The 48 EAs were further randomized in a 2 × 2 factorial design to receive standard treatment coverage (one day visit by treatment team) versus enhanced coverage (two visits to each EA). Randomization of EAs was stratified by district (six EAs per district allocated to either three mass treatments or one).

The PRET trial sample size, 100 children in each of 48 EAs, was estimated in order to power analyses of trachoma outcomes, which were the prevalence of follicular trachoma (TF) and *C. trachomatis* infection in children aged 0–5 year at the three years follow-up survey; no additional power calculations were performed. Anthropometry measures at the three years follow-up survey were specified as secondary outcomes of the trial in April 2011, two months ahead of the three years follow-up survey. Of the children aged 0–5 years randomly selected for measurement of trachoma outcomes at the three years follow-up, those aged 1–4 years were eligible to participate in the anthropometry sub-study.

### Intervention

The intervention was MDA with a single, oral dose of azithromycin given at 20 mg/kg body weight, up to 1 g. Height was used as a proxy for weight [31]. Pregnant women and infants under the age of 6 months were instead offered tetracycline eye ointment for daily use for up to 6 weeks. Treatment was distributed to all 48 EAs at baseline (July-August 2008) and to those 24 EAs assigned to the annual MDA arm at the one and two years follow-up. Treatment was distributed by the Gambia’s National Eye Health Programme as previously described [29]. Coverage was calculated as the proportion of residents present at the time of treatment who were treated.

### Field methods

**Figure 1:**
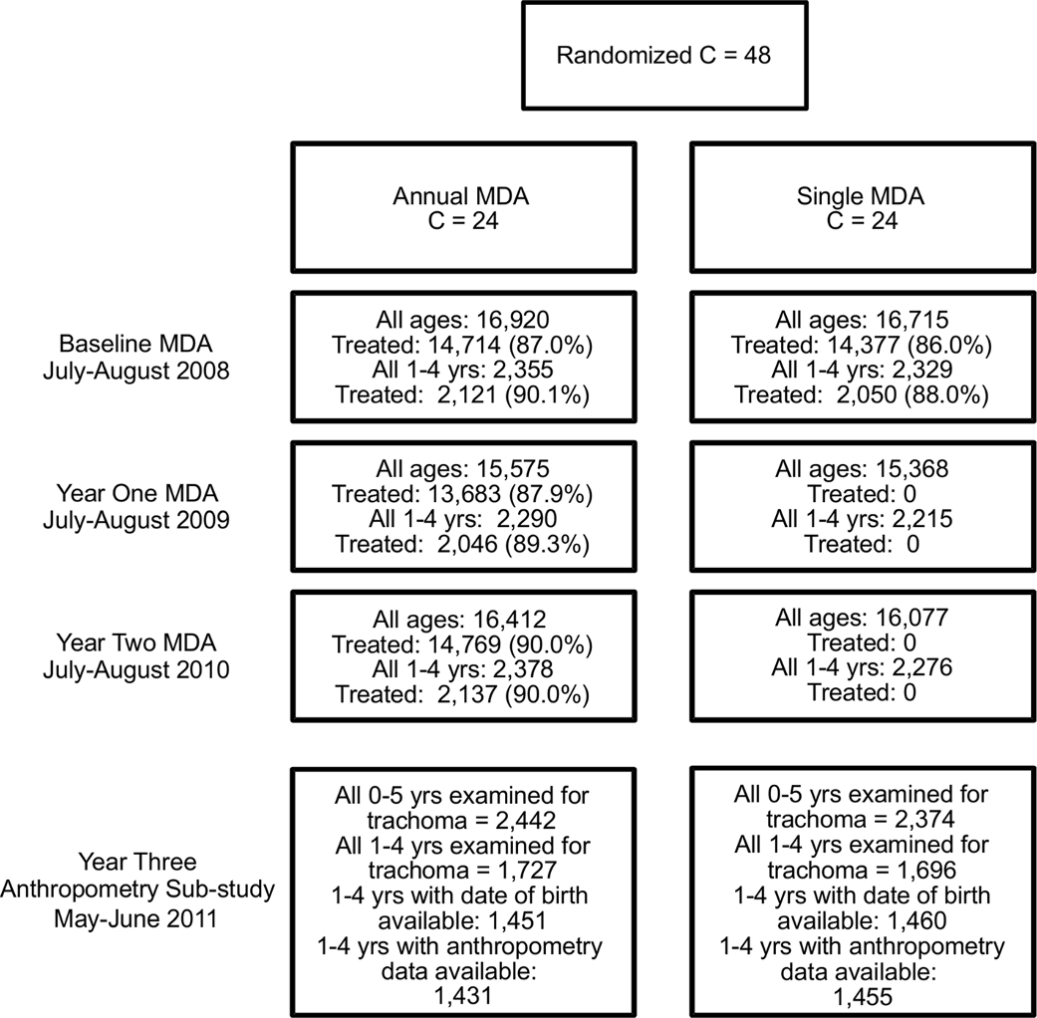
**Study flow.**

Data collection for this ancillary study took place during the three years follow-up survey of the PRET trial and was conducted in May and June 2011, corresponding to three years after treatment in the single MDA arm and one year after the final round of treatment in the annual MDA arm (Figure 1). The PRET trial census data, which was updated every six months and included information on child name, age, sex and parents, was used to select children for measurement of trachoma outcomes and to provide the basis for data on coverage of mass treatment.

Because data collection took place one year after the final round of azithromycin treatment in the annual MDA arm, children under one year of age in this arm would not have received treatment. We therefore focused our study on children aged 1–4 years; height and weight measurements were collected from those children, aged 1 to 4 years, who were selected for measurement of trachoma outcomes by the PRET trial. Eligible children were: resident in the EA, did not have a condition precluding ocular examination and had a guardian willing to provide consent. Weight was measured using Seca electronic scales (Seca gmbh & co. kg., Hamburg, Germany) to within 0.1 kg. Children who were unable to stand on the scales unaided were held by a parent or guardian using the tare function to subtract the weight of the adult. Height was measured with a Seca length board (for those children unable to stand unaided) or a Seca Leicester height measure (Seca gmbh & co. kg., Hamburg, Germany) to within 0.1 cm. Individuals taking height and weight measurements participated in standardized training, conducted by a research clinician, prior to data collection and were blind to the treatment arm of each community. Community ophthalmic nurses and medical doctors treated incidental medical conditions in the field. All visibly malnourished children were referred to the local health centre for treatment.

### Statistical methods

Data were double entered into bespoke databases created in Microsoft Access. Analyses were performed using Stata, version 12 (StataCorp LP, College Station, Tx, USA).

Community and household attributes at baseline were summarized by arm by calculating mean summary measures of cluster level attributes and then calculating the mean of cluster level summary measures for each arm. Characteristics of children included in the anthropometric study at the three years follow-up were summarized as the overall number and percentage of children with each characteristic by study arm.

Height-for-age (HAZ), weight-for-age (WAZ) and weight-for-height (WHZ) *z* scores were calculated for children aged 1–4 years using user-written WHO programs for Stata software (http://www.who.int/childgrowth/en/). Children with a HAZ, WAZ or WHZ <-2 were classified as stunted, underweight or wasted, respectively [32].

Continuous *z* score outcome measures were summarized as the overall mean and standard deviation (SD) by arm. The prevalence of wasting, stunting and underweight was summarized using the overall number and percent of children with each outcome by arm. Only children aged 1–4 years for whom date of birth data were available were included in the analyses.

Comparisons were made between anthropometric outcomes of children residing in the annual MDA arm and those residing in the single MDA arm to determine whether repeated treatment was associated with improved growth. The primary analysis on this open cohort was by intention-to-treat (ITT). A secondary ITT analysis was conducted in a nested, closed cohort of children enrolled in the PRET trial at baseline, who were, in theory, eligible for all three repeated treatments (dependent on allocation of their community). It was hypothesised that if there were a beneficial impact of repeated annual MDA on anthropometric outcomes, this secondary analysis would show an emphasised effect. For each analysis, the same approach was taken; each of the six outcomes (WAZ, HAZ, WHZ and prevalence of wasting, stunting and underweight) was compared by arm (annual MDA versus single MDA) using random effects linear regression to account for between-community and between-household variation as appropriate. All children aged 1–4 years with available data were included. Adjustments were made in all models *a priori* for latrine access (yes versus no), time to water (<15 minutes versus ≥15 minutes) and district. In order to explore the hypothesis that severely malnourished children would benefit more from treatment, the distribution of *z* scores by arm was formally tested using a Kolmogorov-Smirnov test.

## Results and discussion

### Study profile

The PRET trial profile is shown in Figure 1. All 48 EAs received mass treatment at baseline (July to August 2008); treatment coverage in 1–4 year olds ranged from 67.4 to 98.9% at the EA level in both arms. Two further rounds of mass treatment were carried out at years one (July to August 2009; EA coverage 67.9 to 100%) and two (July to August 2010; EA coverage 65.1 to 98.9%) in those 24 communities randomized to the annual MDA arm. Anthropometric indices were measured in all 48 EAs one year following the final round of MDA (May to June 2011) to explore the hypothesis that better indices would be seen in children residing in EAs that received three rounds of MDA versus EAs that had received a single round.

Community and household attributes at baseline, including access to water, access to latrines and level of household-head education, were balanced by arm (Table 1). Three years post-baseline, these attributes were similar to baseline and by arm (data not shown).

**Table 1:** **Characteristics of study population**

		Annual MDA	Single MDA
**Baseline census:**			
Number of clusters (EA) randomized		24	24
EA population size (all ages), mean (SD)*		705 (239.3)	696 (184.5)
EA population size (1–4 years), mean (SD)*		98 (43.4)	97 (28.5)
Household head years of education, mean (SD)*		0.61 (0.79)	0.51 (0.66)
Household water source >15 minutes away, mean (SD)*		12.0 (14.7)	12.6 (20.2)
Household latrine access, mean (SD)*		90.0 (12.4)	92.0 (9.14)
**Year three follow-up:**			
Number of clusters		24	24
Total number of children aged 0 to 5 years randomly sampled and examined for trachoma outcomes		2442	2374
Number of children aged 1–4 years examined for trachoma outcomes		1727	1696
Number of children aged 1–4 years examined for trachoma outcomes and with anthropological measures		1431	1455
District	Foni Bintang	295 (20.6)	357 (24.5)
	Foni Kansala	288 (20.1)	327 (22.5)
	Lower Baddibu	443 (31.0)	365 (25.1)
	Central Baddibu	405 (28.3)	406 (27.9)
Age (years)	1	386 (27.0)	354 (24.3)
	2	369 (25.8)	408 (28.0)
	3	362 (25.3)	347 (23.9)
	4	314 (21.9)	346 (23.8)
Sex	Male	741 (51.8)	765 (52.6)
	Female	690 (48.2)	690 (47.4)
Active trachoma	No	1388 (97.0)	1408 (96.8)
	Yes	43 (3.0)	47 (3.2)

*data summary measures are mean (SD) of cluster proportions or means by arm.

A total of 4,816 children aged 0 to 5 years were selected for measurement of trachoma outcomes at the three years follow-up survey of the PRET trial (Table 1). Of these, 3423 children aged 1–4 years were eligible for the anthropometric analysis and within this group, 2886 children (approximately 85% overall and by arm) had data available for height, weight and date of birth (Figure 1, Table 1). Children excluded due to missing data did not differ systematically from the remaining children and data were therefore assumed to be missing at random. Final analyses included 1431 children in the annual MDA arm and 1455 children from the single MDA arm.

Child level attributes three years post-baseline were balanced by arm (Table 1) with respect to distribution of children by age in years, sex and district of residence.

### Comparisons of three annual rounds of MDA versus a single round

A comparison of mean *z* scores of children residing in EAs randomized to three rounds of MDA versus those residing in EAs where a single round of MDA was carried out revealed no significant differences; the mean *z* scores were approximately -1.3 (HAZ), -1.1 (WAZ) and -0.6 (WHZ) in both arms (Table 2). In agreement with these results, no statistically significant decreases in the prevalence of stunting (25% in each arm), underweight (17-18% per arm) and wasting (8% in each arm) were noted for the annual MDA versus single MDA arms (Table 2).

**Table 2:** **Comparison of outcome measures in all children aged 1 to 4 years in annual MDA versus single MDA trial arms**

Outcome	Annual MDA N = 1431	Single MDA N = 1455	Adjusted* coefficient (95% CI), p-value
WHZ, mean (SD)	-0.60 (1.01)	-0.60 (1.06)	-0.02 (-0.10 to 0.06), 0.651
HAZ, mean (SD)	-1.28 (1.13)	-1.35 (1.21)	-0.07 (-0.19 to 0.05), 0.257
WAZ, mean (SD)	-1.11 (0.96)	-1.15 (1.00)	-0.05 (-0.14 to 0.04), 0.264
			Adjusted† OR (95% CI), p-value
Wasted, n (%)	114 (8.0)	120 (8.3)	1.07 (0.81 to 1.40), 0.642
Stunted, n (%)	343 (24.0)	371 (25.5)	1.12 (0.90 to 1.41), 0.303
Underweight, n (%)	242 (16.9)	264 (18.1)	1.10 (0.89 to 1.37), 0.380

OR = odds ratio, CI = confidence interval.

*adjusted for between-household variation, between-EA variation, latrine access, time to water and district. P-values are from likelihood ratio tests comparing adjusted models with and without trial arm (annual MDA versus single MDA).

† adjusted for between-household variation and between-cluster variation as appropriate and *a priori* for latrine access, time to water and district. P-values are from likelihood ratio tests comparing adjusted models with and without arm.

By approximately two years of age, growth faltering in Gambian children slows, consistent with global trends [33]. One may therefore expect younger children to benefit more from azithromycin treatment than older children. However, our data do not show any evidence of interaction between arm and age group for any of the *z* score outcomes (data not shown).

We also examined potential shifts in the distribution of *z* scores, hypothesising that severely malnourished children would benefit more from treatment than other children. However, analyses found no distributional change for any of HAZ, WAZ or WHZ scores between treatment arms (Kolmogorov-Smirnov test for equality of distributions: WHZ, p = 0.470; HAZ, p = 0.228; WAZ, p = 0.217).

As the majority of children in the single MDA arm would not have received azithromycin treatment (treatment took place three years prior to data collection) and as children less than 3 years of age in the annual MDA arm would have received a varying number of treatment doses (one or two), we also performed an analysis restricted to those children, in both arms, who were present at the baseline census of the PRET trial. ITT analyses of this closed cohort showed no difference between arms for any of the six measures (Table 3).

**Table 3:** **Comparison of outcome measures in children enrolled in the PRET trial at baseline, in annual MDA versus single MDA trial arms (aged 3 to 4 years)**

Outcome	Annual MDA N =569	Single MDA N =577	Adjusted* coefficient (95% CI), p-value
WHZ, mean (SD)	-0.69 (0.92)	-0.67 (0.94)	0.02 (-0.09 to 0.13), 0.756
HAZ, mean (SD)	-1.15 (1.01)	-1.16 (1.04)	-0.01 (-0.16 to 0.14), 0.885
WAZ, mean (SD)	-1.13 (0.88)	-1.13 (0.87)	0.01 (-0.10 to 0.12), 0.859
			Adjusted† OR (95% CI), p-value
Wasted, n (%)	40 (7.0)	42 (7.3)	1.06 (0.68 to 1.67), 0.796
Stunted, n (%)	107 (18.8)	107 (18.5)	0.98 (0.72 to 1.35), 0.912
Underweight, n (%)	90 (15.8)	90 (15.6)	0.96 (0.70 to 1.32), 0.804

OR = odds ratio, CI = confidence interval.

* adjusted for latrine access, time to water, and district. P-values are from random effects linear regression models.

† adjusted for latrine access, time to water and district. P-values are from comparing random effects logistic regression models with and without trial arm.

### Limitations

This study has a number of limitations. Due to the design of the PRET trial, there were no untreated study EAs, which would have enabled a comparison of one and three rounds of MDA to no treatment. This would have been of interest particularly as the greatest decreases in child mortality reported in the Ethiopian studies were seen when comparing treated children to untreated children [10, 11]. The length of time since MDA differs in each study arm; the cross-sectional survey took place one or three years after the final round of MDA in the annual MDA and single MDA arms respectively. The complexity of the cluster randomised design and opportunities for multiple treatments (and hence multiple missed treatments through non-participation) meant that evaluation of the effect of treatment at the individual level was not possible. Baseline anthropometric data from study villages before the commencement of MDA are also lacking. While close to 1,700 children aged 1–4 years were recruited into each arm of the study, *z* scores could not be calculated for approximately 15% of them, as dates of birth were not available, thereby reducing power. Three individuals took height and weight measurements and there may have been small systematic differences between measurers although standardized training of all workers was undertaken prior to the study to minimise this effect.

## Conclusions

MDA with azithromycin for trachoma control aims to reduce the community reservoir of *Chlamydia trachomatis* infection thereby interrupting transmission. However, the broad-spectrum activity of azithromycin may bring additional benefits. For example, MDA with azithromycin given for trachoma reduces the prevalence of nasopharyngeal *Streptococcus pneumonia* carriage, which in turn has the potential to reduce the community burden of respiratory disease. As infectious disease can drive malnutrition [34], we hypothesized that children residing in Gambian communities were three rounds of MDA were carried out would have improved anthropometric indices in comparison to children residing in communities where a single round of MDA was given, via a potential reduction in community burden of infectious disease.

Although azithromycin MDA has been linked with reductions in morbidity and mortality in other settings and in spite of the PRET trial having achieved high treatment coverage [35], our study did not find evidence that three annual rounds of MDA improved anthropometric indices compared to a single round. While the study design and sample size may have limited the ability to detect a difference, MDA given at yearly intervals may also not be sufficient to impart lasting improvements on growth. The PRET trial in Niger has compared anthropometric indices of children in communities randomized to annual versus biannual treatment thereby comparing a single dose with two doses given at 6-month intervals [36]. In that study, small improvements for all *z* scores, including mid-upper arm circumference, were noted in communities randomized to the biannual treatment arm although they were not statistically significant [36]. This does not provide evidence that MDA given biannually has a long-term impact on growth. The reported reductions in incidence of diarrhoeal and respiratory disease following MDA have been shown to last approximately three months, after which time, incidence rates are similar to that seen in untreated villages [19, 20]. To have a sustained impact, MDA may need to be given at more frequent intervals however frequent treatment has been associated with an increase in the prevalence of carriage of macrolide resistant bacteria in the community [37] and, as such, requires careful monitoring.

It may also be that the mechanism by which azithromycin acts (i.e. antibacterial) is not sufficient to drive significant improvements in anthropometric indicies in the Gambian setting. In The Gambia, diarrhoeal disease has been associated with both height and weight faltering [14, 15, 38] but a recent study has shown that much of the burden of diarrhoeal disease in rural Gambia today is attributable to viral and protozoan infections (primarily rotavirus and *Cryptosporidium*), which cannot be treated with azithromycin [14]. Although data are limited, respiratory disease in The Gambia has been associated with weight faltering [15, 38]. However, vaccine coverage to protect against *Streptococcus pneumoniae* and *Haemophilus influenzae* serotype B is high in the country [39] and data suggest the greater burden of acute lower respiratory disease in young Gambian children is due to viral (RSV and influenza) rather than bacterial infections [40], which again are not susceptible to azithromycin treatment.

Our findings do not indicate MDA with azithromycin for trachoma control has improved anthropometric indices of Gambian children. Furthermore, data available at present do not suggest improved nutritional status as a mechanism behind the reduction in child mortality attributed to intermittent MDA with azithromycin.
